# Health Extension Workers Improve Tuberculosis Case Detection and Treatment Success in Southern Ethiopia: A Community Randomized Trial

**DOI:** 10.1371/journal.pone.0005443

**Published:** 2009-05-08

**Authors:** Daniel G. Datiko, Bernt Lindtjørn

**Affiliations:** 1 Centre for International Health, University of Bergen, Bergen, Norway; 2 Southern Nations, Nationalities, and Peoples' Regional Health Bureau, Awassa, Ethiopia; National Institute for Infectious Diseases (INMI) L. Spallanzani, Italy

## Abstract

**Background:**

One of the main strategies to control tuberculosis (TB) is to find and treat people with active disease. Unfortunately, the case detection rates remain low in many countries. Thus, we need interventions to find and treat sufficient number of patients to control TB. We investigated whether involving health extension workers (HEWs: trained community health workers) in TB control improved smear-positive case detection and treatment success rates in southern Ethiopia.

**Methodology/Principal Finding:**

We carried out a community-randomized trial in southern Ethiopia from September 2006 to April 2008. Fifty-one kebeles (with a total population of 296, 811) were randomly allocated to intervention and control groups. We trained HEWs in the intervention kebeles on how to identify suspects, collect sputum, and provide directly observed treatment. The HEWs in the intervention kebeles advised people with productive cough of 2 weeks or more duration to attend the health posts. Two hundred and thirty smear-positive patients were identified from the intervention and 88 patients from the control kebeles. The mean case detection rate was higher in the intervention than in the control kebeles (122.2% v*s* 69.4%, p<0.001). In addition, more females patients were identified in the intervention kebeles (149.0 *vs* 91.6, p<0.001). The mean treatment success rate was higher in the intervention than in the control kebeles (89.3% *vs* 83.1%, p = 0.012) and more for females patients (89.8% *vs* 81.3%, p = 0.05).

**Conclusions/Significance:**

The involvement of HEWs in sputum collection and treatment improved smear-positive case detection and treatment success rate, possibly because of an improved service access. This could be applied in settings with low health service coverage and a shortage of health workers.

**Trial Registration:**

ClinicalTrials.gov NCT00803322

## Introduction

Each year, more than nine million new cases of tuberculosis (TB) occur and about two million people die of TB. As a result of the interaction between TB and human immunodeficiency virus (HIV) infection, TB incidence is rising in sub-Saharan Africa. It has also led to an increase in drug resistance and poor treatment outcomes [1].

Information from South India shows that directly observed treatment, short-course (DOTS) reduces TB incidence [2]. However, in many other countries, the case detection rates are too low to reduce the incidence of TB. The main obstacles are low health service coverage, shortage of health workers and poor programme performance [3].

Epidemiological models show that active case finding might reduce TB incidence and avoid TB deaths. Although active case finding is effective in contact tracing on a small scale, high cost and poor treatment adherence limit its use [4], [5]. We therefore need alternative methods to improve TB case finding.

In Ethiopia, the National TB and Leprosy Control Programme (NTLCP) started to implement DOTS in 1992. NTLCP is responsible for policy formulation, resource mobilisation, monitoring and evaluation. Under the NTLCP, three levels of function exist in the regions, zones and districts for coordinating TB control activities. TB control is also integrated into the general service at health facilities. The district TB programme coordinator is responsible for supervision of the general health workers involved in patient care in hospitals and health centres. However, community DOTS was not started.

Ethiopia has the seventh highest TB burden in the world. In 2006, the estimated number of new smear-positive cases was 168 per 10^5^ for Ethiopia. Unfortunately, the case detection rate was 27%, far below the target [6]. In 2004, the government of Ethiopia launched a community-based initiative to provide essential health services to the community under a health extension programme (HEP) to ensure equitable access to health services. The aim of the HEP is to prevent major communicable diseases and promote health in the community. A new cadre of community level health workers, health extension workers (HEWs), was trained for 1 year at an undergraduate level. With the aim of preventing major communicable diseases, HEWs are trained on how to identify and refer TB suspects, trace defaulters, and provide treatment and health education [6], [7]. However, their role in TB control has not been evaluated. The aim of the present study was to establish whether involving HEWs in TB control improved smear-positive case detection and treatment success rates in southern Ethiopia.

## Methods

The protocol for this trial and supporting CONSORT checklist are available as supporting information; see Checklist S1 and Protocol S1.

### Study area and population

This study was conducted in Dale and Wonsho, rural districts of Sidama zone in southern Ethiopia from September 2006 to April 2008. There were 51 kebeles (lowest administrative units) in the two districts. Fifty-five per cent of the population live within two-hour walking distance of health facilities. There were 21 health posts (operational unit for HEWs), two health stations, two nucleus health centres (health stations upgrading to health centres) and one health centre. Three health facilities (one health centre and two health stations) conducted sputum microscopy, and DOT was provided in the health centre, nucleus health centres and health stations. None of the health posts provide DOT.

### Health service and HEP

The Government of Ethiopia has a four-tier health service, and the lowest level is a primary health care unit (a health centre and five satellite health posts). On average, a health post serves a kebele with 5000 people. The health policy focuses on provision of preventive and promotive health care to the population under the HEP, which involves prevention and control of diseases, including TB. The local health authorities in consultation with kebele leaders select two female residents, who have completed tenth grade, from each kebele. The women receive training for 1 year and are placed as HEWs in their respective kebele. They receive a salary from the government and they are accountable to the health centre [7].

### Participants

### TB case finding and treatment outcome

#### Case finding

TB suspects, who had cough for two weeks or more, were referred for further investigations. A smear-positive pulmonary TB case was defined by two positive sputum smears or one positive smear and x-ray findings consistent with active TB.

#### Treatment regimen and duration

The treatment regimen for new smear-positive cases consisted of two months intensive phase treatment with ethambutol, rifampicin, isoniazid and pyrazinamide followed by continuation phase treatment for 6 months with ethambutol and isoniazid. For children, in the continuation phase, ethambutol/isoniazid was replaced by rifampcin/isoniazid for 4 months. Follow-up sputum smear examination was done at the end of 2, 5 and 7 months treatment.

#### Treatment outcome

A patient with at least two negative smears including that at 7 months was reported as cured. A patient who finished the treatment but did not have the 7-month smear result was reported as treatment completed. If a patient remained or became smear-positive at the end of 5 months or later, he/she was reported as treatment failure. A patient who missed treatment for eight consecutive weeks after receiving treatment for at least 4 weeks was reported as a defaulter. A patient who was transferred to another district after receiving treatment for at least 4 weeks and whose treatment outcome was not reported to the referring district was reported as transferred out. A patient who died while on treatment was reported as dead irrespective of the cause of death [8].

#### Ethics

We obtained ethical clearance from the Ethical Review Committee of the Regional Health Bureau in southern Ethiopia. We obtained permission from TB programme managers and kebele leaders after discussing with them community-based TB care. TB patients were enrolled after giving informed consent after explaining the aim of the study and the right to refuse or to withdraw from the study. HIV testing was not offered to TB patients because of the unavailability of HIV testing and treatment in the study area at the time the study was conducted.

### The intervention

#### Training on how to identify TB suspects and administer DOT

We trained health workers, laboratory technicians and HEWs for 2 days. The training focused on symptoms and transmission of TB, how to identify TB suspects, how to collect, label, store and transport sputum specimens, administer DOT, and follow patients during treatment. The messages and the content of our training were similar to the curriculum of training HEWs. HEWs, in the in the intervention kebeles, received on job training about how to collect sputum samples and support patients to adhere to treatment. HEWs collected sputum specimens once a month. An ice box was used to keep the sputum specimens in the health post and during their transportation on foot to diagnostic units. The intervention included sputum collection and providing DOT.

During health education sessions at health posts, HEWs informed people living in the kebele about TB and advised them to come to a health post if they had productive cough of 2 weeks or more duration. TB suspects who came to the health posts were told about community-based TB care. HEWs collected spot-morning-spot sputum specimens, and labelled and transported them to the diagnostic units every month for examination for acid-fast bacilli by direct microscopy. Smear-positive patients in the intervention kebeles received standard DOTS under the direct observation of HEWs. TB patients visited health posts daily during the intensive phase and once a month in the continuation phase.

### Control kebeles

#### Identifying TB suspects and DOT administration

HEWs in the in the control kebeles did not received on job training about how to collect sputum samples and how to support patients to adhere to treatment. However, they provided health services, including health education about TB, to the people living in their kebeles. TB suspects presented themselves to diagnostic units. However, the health workers from health facilities were trained as they provided the service to intervention and control kebeles. Smear-positive patients in the control kebeles received standard DOTS were treated under the direct observation of general health workers at health centres. TB patients visited health centres and health stations daily during the intensive phase and once a month in the continuation phase.

#### Objective

the objective of the study was to investigate whether involving HEWs in TB control improves the case detection and treatment success rate in southern Ethiopia

### Outcome variables

#### Case detection rate

the number of new smear-positive cases detected divided by the estimated number of incident smear-positive cases, expressed as a percentage.

#### Treatment success rate

cure or treatment completion rate was calculated as the number of patients cured or treatment completed divided by the total number of patients reported expressed as a percentage. Treatment success rate (TSR) was the sum of cure and treatment completion rate.

### Sample size calculation

The sample size was calculated based on a difference in effect size of 30%, power of 80%, 95% significance level, and coefficient of variation of 0.25. Based on the average annual smear-positive case detection rate (CDR) of 41% (unpublished review of three years of DOTS in the study area; the national CDR was 29%), we calculated the number of clusters required per group with 30% contingency. Based on the principle of allocating an unequal number of clusters for randomization [9], we allocated 30 kebeles to the intervention and 21 kebeles to the control group.

### Randomization: generation and implementation

**Figure 1:**
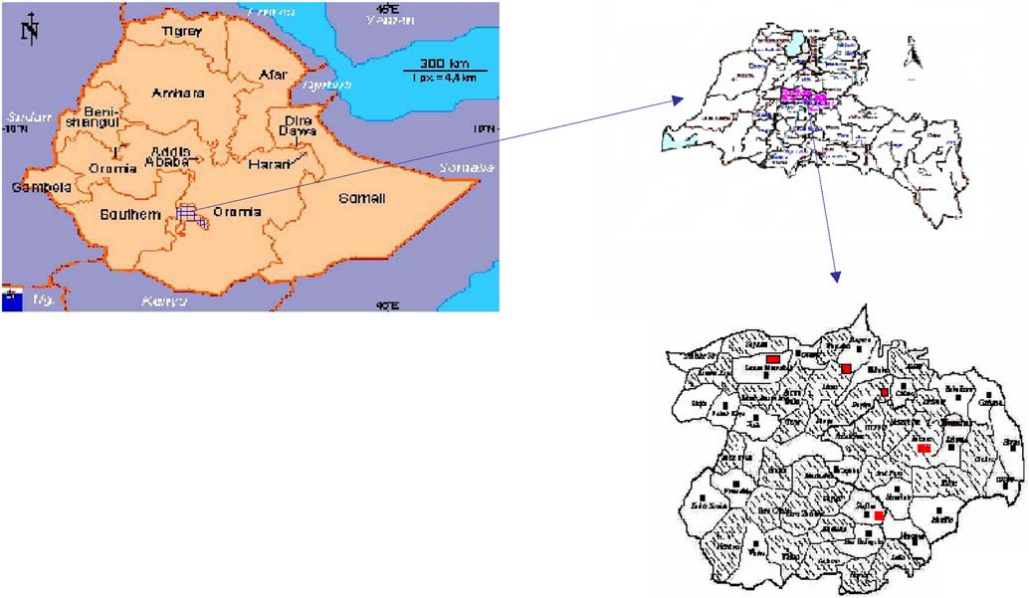
Map of the study area in Sidama zone in south Ethiopia. Shaded area - Intervention kebeles. White area with black box - Control kebeles. Red box - Health centers and health stations.

Before starting the intervention, we explained the aim of the study to the programme coordinators of the districts and health facilities. After we obtained their consent, we used the list of kebeles in the two districts and randomly allocated them to intervention and control groups using a table of random numbers (Figure 1).

### Blinding

Neither the general health workers nor TB programme managers were blinded to the allocation. Although we did not blind the laboratory technicians, they were not informed whether the sputum specimens were from the intervention or control kebeles.

### Data collection

TB case finding and treatment outcome data were collected from TB registers at health facilities and districts. The information collected included date, age, sex, address, TB classification, smear results and treatment outcome using the official reporting system of the NTLCP.

### Statistical analysis

We used Microsoft excel and SPSS for Windows 14 (SPSS Inc, Chicago, USA) for data entry and analysis. We analysed the data on the basis that all TB patients in the intervention kebeles intended to use community-based case finding and treatment. We described the patients by age, sex, season and treatment outcome. We calculated summary values of case detection and treatment success rates for each kebeles. We used independent sample *t* test, weighted by cluster size, to compare the mean CDR and TSR using kebele as a unit of analysis. This is robust for cluster level analysis of binary outcomes [9]. The intra-cluster correlation coefficient was calculated using one-way analysis of variance [10], [11].

## Results

### Participants flow, recruitment and number analysed

**Figure 2:**
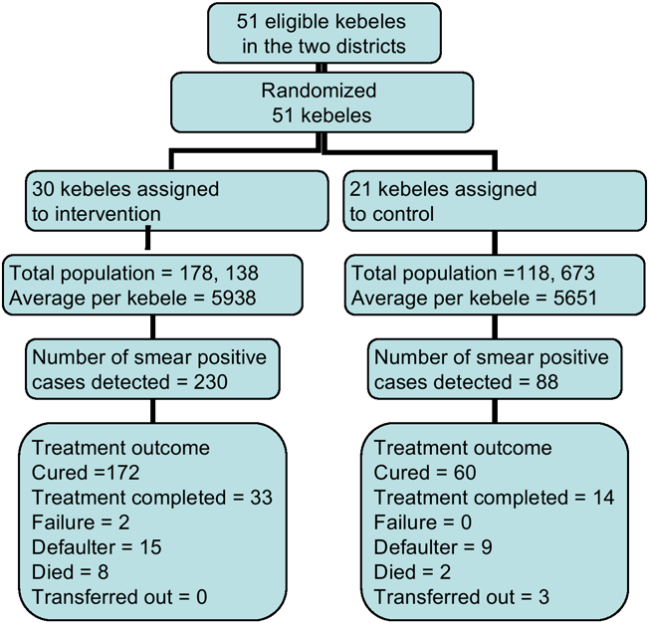
Trial profile for smear-positive TB case finding and treatment outcome.

In a year, the number of pulmonary TB suspects examined was 723 from intervention and 328 from control kebeles. Among these, 230 and 88 smear-positive patients were identified from the intervention and control kebeles, respectively. All the smear-positive patients were analyzed (figure 2).

### Baseline data

Of the 51 kebeles included in the study, 30 were intervention kebeles with a population of 178,138 and mean kebele population of 5938 people, while 21 were control kebeles with a population of 118,673 and mean kebele population of 5651 people. 53.4% (123/230) of patients from intervention and 42% (37/88) from control kebeles were female (Table 1).

**Table 1:** The baseline characteristics of the study area and smear-positive tuberculosis cases of southern Ethiopia 2006/07.

Variable	Intervention	Control
**Communities**		
Number of clusters	30	21
Study population	178, 138	118, 673
Male	91,206	63,464
Female	86,932	55,209
Mean kebele population	5938	5651
Male	3040	3022
Female	2898	2629
**Smear-positive TB patients**		
Mean age (SD)	29 (13)	26 (11)
Male	29 (13)	26 (13)
Female	29 (13)	24 (8)
Number (%) of TB cases by sex		
Male	107 (46.6)	51 (58)
Female	123 (53.4)	37 (42)
Number (%) of TB cases by age(in years)		
≤14	23 (10.0)	9 (10.3)
15–24	63 (27.4)	34 (39.1)
25–34	72 (31.3)	28 (32.2)
35–44	58 (25.2)	13 (14.9)
45–54	14 (6.1)	3 (3.4)
Number (%) of TB cases by season		
Spring	55 (23.9)	29 (33.0)
Winter	69 (30.0)	18 (20.4)
Autumn	45 (19.6)	22 (25.0)
Summer	61 (26.5)	19 (21.6)

### Outcomes and estimation

Patients from control kebeles were younger than those from intervention kebeles (26 *vs* 29 years, p = 0.011). The mean CDR was higher in intervention kebeles (122.2% *vs* 69.4%, p<0.001) and for female patients (149.0% *vs* 91.6%, p<0.001) (Table 2).

**Table 2:** Case detection rates of smear-positive tuberculosis cases in southern Ethiopia, 2006/07.

Variable	Intervention	Control	Mean difference (95%CI)	P - value	ICC*
**CDR** † **(%)**	122.2	69.4	52.8 (39.8–65.4)	<0.001	0.00052
Male	112.6	86.0	26.6 (7.1–46.0)	0.008	0.00039
Female	149.0	91.6	57.4 (31.9–82.9)	<0.001	0.00073
**For ≤14 years (%)**	82.9	31.9	50.9 (26.8–75.2)	<0.001	0.00049
Male	69.8	44.1	25.6 (5.4–45.9)	0.018	0.00024
Female	115.6	45.5	70.1 (29.–110.6)	0.002	0.00065
**For >14years (%)**	193.7	118.2	75.5 (55.6–95.5)	<0.001	0.00060
Male	184.7	149.4	35.3 (4.2–66.5)	0.027	0.00038
Female	235.9	170.9	64.9 (15.6–114.4)	0.011	0.00098
**By season (%)**					
Spring	227.1	104.2	122.9 (70.9–174.9)	<0.001	0.00136
Winter	138.5	80.8	57.7 (36.2–79.2)	<0.001	0.00013
Autumn	136.2	114.2	21.8 (−19.4–62.9)	0.294	0.00061
Summer	169.5	87.4	82.0 (50.9–113.1)	<0.001	0.00069

* ICC - intraclass correlation coefficient.

† CDR - case detection rate.

Among the 230 patients from the intervention kebeles, 172 (74.8%) were cured, 33 (14.3%) completed treatment, eight (3.5%) died, two (0.9%) had treatment failure, 15 (6.5%) defaulted and no patient was transferred out. Of the 88 patients in the control kebeles, 60 (68.2%) were cured, 14 (15.9%) completed treatment, two (2.3%) died, nine (10.2%) defaulted, three (3.4%) were transferred out, and none had treatment failure (Figure 2). The mean TSR was higher in the intervention than control kebeles (89.3% *vs* 83.1%, p = 0.012). Similarly, the mean TSR for females was higher in the intervention than control kebeles (89.8 *vs* 81.3%, p = 0.05) as shown in Table 3.

**Table 3:** Treatment success rates of smear-positive tuberculosis cases in southern Ethiopia, 2006/07.

Variable	Intervention	Control	Mean difference (95%CI)	P - value	ICC*
**TSR** † **for all (%)**	89.3	83.1	6.2 (1.4–10.9)	0.012	0.00052
Male	87.0	84.3	2.7 (−4.8–0.2)	0.471	0.00017
Female	90.9	81.1	9.9 (1.6–18.2)	0.202	0.00035
**For ≤14 years (%)**	91.3	88.9	2.4 (−17.4–22.2)	0.805	0.00028
Male	87.5	75.0	12.5 (−64.6–89.6)	0.657	0.00003
Female	93.3	100	−6.7 (−31.4–18.0)	0.578	0.00017
**For >14years (%)**	88.9	80.8	8.2 (2.6–13.8)	0.005	0.00029
Male	88.0	80.4	7.6 (−1.5–16.6)	0.101	0.00009
Female	89.8	81.3	8.6 (−0.1–17.3)	0.05	0.00019
**By season (%)**					
Spring	89.1	89.6	−0.6 (−10.0–8.9)	0.906	0.00024
Winter	84.1	93.8	−9.9 (−20.9–1.6)	0.090	0.00004
Autumn	73.3	68.2	5.2 (−15.4–25.8)	0.619	0.00013
Summer	83.6	89.5	−5.9 (−22.4–10.7)	0.470	0.00018

* ICC - intraclass correlation coefficient.

† treatment success rate.

## Discussion

### Interpretation and overall evidences

We showed that involving HEWs in TB control improved the smear-positive CDR and TSR in the intervention kebeles. Both the CDR and TSR were higher for female patients in the intervention kebeles.

DOTS uses passive case finding to detect TB cases, through health education and tracing contacts of index cases [6]. However, decades after implementing the strategy, smear-positive CDR has remained far below the target. In particular, the trend in CDR was consistently low for women, to the extent that passive case finding seems to favour men [12], [13], [14], [15]. The reasons are low health service coverage, shortage of trained health workers and poor health seeking behaviour [3], [16], [17]. Alternatively the advantage of active case finding in improving case detection is limited due to the associated high cost in resource-constrained settings [5], [18]. Moreover, neither rapid community surveys [19], [20] nor community DOT [21], [22] seems to improve CDR.

In our study, community-based case finding significantly improved the CDR for all age groups more for women than for men. The increase in CDR was lower for children compared to those aged 15 years and above. This could be explained by an inability to produce sputum specimens, low disease burden, or the low number of children enrolled in the study [23]. Patients from the intervention kebeles were older than those from control kebeles for both sexes. This may have been caused by poor access, poverty, and low health seeking behaviour that might have hindered them from coming to the health facilities.

Routine surveillance reports have repeatedly shown higher CDRs for men than women [24]. However, in our study, the CDR was higher for females in the intervention group. This could be explained by the improved geographic and socioeconomic access to the service as sputum collection was done in the intervention kebeles. As expected, the number of TB cases detected was greater than that estimated. This may have resulted from underestimation of TB incidence as reported from Myanmar [8], the backlog of TB cases that were not reached by the health service [19], [24], or underestimation of the population in the study area. Further study is required to determine the magnitude of TB in the community.

Studies have shown that using different treatment supervisors for DOT has improved the TSR for passively detected TB cases [25], [26], [27]. However, poor treatment adherence remains a challenge for patients identified by active and enhanced case finding [28]. In our study, decentralisation of the treatment to the kebele improved the TSR for TB patients detected by enhanced case finding. Similar to CDR, the TSR was higher for women aged above 14 years because of improved access created by DOT provision in the kebele.

Our findings suggest seasonal variation in CDR and TSR. In the intervention kebeles, the rates peaked in spring (September–November) and winter (December–February) possibly as a result of the economic gain from the harvest in spring. However, in the intervention and control kebeles, the rates were low in autumn (March–May) when farmers prepare for the farming season, and this was followed by another peak in early summer (June–August). Previous studies have suggested that overcrowding and staying indoors during the rainy season favour transmission of TB, which results in greater seasonal variation in children [29], [30], [31]. In our setting, further study is required to establish more about the seasonal variation and its associated factors.

Although cluster randomized controlled trials are considered valid studies, their methodological limitations should be addressed. The baseline demographic and clinical characteristics were similar in the two groups. We kept potential for bias to a minimum by comparing and analysing information from complementary sources. Although we did not blind the sputum samples sent to the diagnostic units, the laboratory technicians received the standard information for sputum analysis, and were not informed if the sputum specimens were from an intervention or control kebele. In addition, external quality control was performed for the slides examined in health facilities at the Centre for Health and Research Laboratory in the southern Ethiopia. In our sample size calculations, the intraclass correlation was small. The CDR and TSR in the control group was 45% and 83%, respectively, which was similar to the CDR of 40% in the intervention and 42% in the control kebeles, and TSR of 78% in the intervention and 74% in the control kebeles (unpublished review of 3 years of DOTS in the study area), which suggests the completeness of our data collection. However, as control and intervention kebeles were neighbouring each other and health facilities delivered the service to both groups, we cannot rule out the effect of the intervention in the control kebeles. This might have reduced the effect size in the intervention kebeles.

Our intervention used enhanced case finding, a variant of active case finding, in which HEWs encouraged TB suspects to visit health posts for sputum collection, and provided DOT. The strength of the study was that it included a sufficient number of clusters to address an important challenge of DOTS strategy, namely low CDR, and improved treatment adherence of patients identified by enhanced case finding by providing DOT. It also explored a practical way of involving HEWs in TB control under the community-based initiative of HEP in Ethiopia.

### Generalizability

The results of our study could be applied in settings with low health service coverage (low DOTS coverage and limited number of TB laboratories), where HEWs have the first contact with the people to provide health education, and collect and transport sputum specimens to diagnostic units. This makes the service patient-centred, to improve case finding and treatment adherence [22]. Our study area is a densely populated agrarian community, typical of the rural population on the Ethiopian highlands. It could also be applied in areas with a shortage of health workers, especially laboratory technicians, with or without adequate health service coverage. The findings of the study were disseminated to managers of TB programmes in the southern region and at national level. We believe our findings are relevant for policy formulation on community TB care in Ethiopia. With limited health care coverage and shortage of health workers, similar to that in many developing countries, we believe that our findings are applicable to similar settings.

In conclusion, involving HEWs in TB control improved the CDR and TSR for smear-positive patients and females in particular. It could be used as an option to improve the trend in low CDR and provide patient-centred services in high-burden countries. However, the cost-effectiveness of enhanced case finding and treatment outcome needs further study.

## Supporting Information

Checklist S1 CONSORT Checklist
(0.06 MB DOC)

Protocol S1 Trial Protocol
(0.27 MB DOC)
