# Supplementary material for: Health Extension Workers Improve Tuberculosis Case Detection and Treatment Success in Southern Ethiopia: A Community Randomized Trial
Source: PLoS One. 2009 May 8;4(5):e5443. doi: 10.1371/journal.pone.0005443 (PMC2678194; doi:10.1371/journal.pone.0005443)
Supplement: Protocol S1 — Trial Protocol (0.27 MB DOC) [file pone.0005443.s002.doc]

**IMPROVING COMMUNITY based TUBERCULOSIS care IN SOUTHERN ETHIOPIA**

**PI**

**Daniel G. Datiko,**

**PhD candidate**

**Supervisor**

**Prof. Bernt Lindtjørn,**

**Centre for International health**

Bergen University, Norway

[1. Global tuberculosis control programme 1](#__RefHeading___Toc215856073)

[1.1. Global programme outline 1](#__RefHeading___Toc215856074)

[1.2. Global TB disease burden 1](#__RefHeading___Toc215856075)

[1.3 TB control programme in Ethiopia 2](#__RefHeading___Toc215856076)

[1.4. TB programme in southern region 2](#__RefHeading___Toc215856077)

[2. Statement of the problem 2](#__RefHeading___Toc215856078)

[3. Literature review 3](#__RefHeading___Toc215856079)

[4. Reason of the study 8](#__RefHeading___Toc215856080)

[5. Goal of the study 9](#__RefHeading___Toc215856081)

[5.1. General objective of the study 9](#__RefHeading___Toc215856082)

[5.2. Specific Objectives: 9](#__RefHeading___Toc215856083)

[6. Methods 9](#__RefHeading___Toc215856084)

[6.1. Study area and population 9](#__RefHeading___Toc215856085)

[6.2. Study design 10](#__RefHeading___Toc215856086)

[6.3. Permission to continue 13](#__RefHeading___Toc215856087)

[6.4. Ethical clearance 13](#__RefHeading___Toc215856088)

[6.5. Data collection and handling 13](#__RefHeading___Toc215856089)

[6.6. Quality assurance 14](#__RefHeading___Toc215856090)

[6.7. Analysis plan 15](#__RefHeading___Toc215856091)

[7. Project management and work plan 15](#__RefHeading___Toc215856092)

**1. Global tuberculosis control programme**

**1.1. Global programme outline**

Mycobacterium tuberculosis has infected humans for thousands of years. The disease, tuberculosis (TB) killed many patients[1]. However, even before introducing antiTB drugs, TB prevalence decreased in developed countries because of improved socio-economic conditions. Unfortunately, the situation worsened globally because of lack of enough control measures.

The World Health Organization (WHO) prioritizes TB control because of the high disease load, feasibility to carry out BCG vaccination and antiTB treatment. These raised the expectation for the coming up with effective strategy for TB prevention and control. With the introduction Rifampicin, Directly observed short course therapy (DOTS) started as a strategy with five parts.[2] These are government commitment to ensure TB control, case detection by sputum smear microscopy among self-reporting symptomatic patients, standardized short course chemotherapy using of six to eight months treatment regimens, regular and uninterrupted supply of all essential antiTB drugs, and standardized recording and reporting[3]

TB control programme started as a vertical programme. This approach was successful in developed countries where it was possible to undergo mass intervention. In developing countries, the approach worked well initially as the programme had its lowest coverage. However, with further programme expansion, it became difficult to oversee the programme from a centralized structure. This led to integrating the control programme into the general health service. TB control programme integrated diagnostic service and treatment units followed by decentralization of executive roles. Given the size of the TB problem, shortage of staff limits prevention and control of TB. Therefore, involvement of private-for-profit organizations, non-government organizations, community health workers (CHWs) and volunteers is needed.[2]

**1.2. Global TB disease burden**

In 2005, 15.4 million TB cases were reported globally. Among 8.8 million people were new TB cases of which 3.9 million were smear positive. The smear positive case detection rate is 42 %. The treatment success rate reached 82% and remained unchanged since then. About 1.7 million people died of TB in the same year. Ninety eight percent of TB deaths occur in the developing countries, mainly affecting young adults. It is the leading cause of death among young women in Africa. If left unchecked, with in 20 years, TB will kill about 35 million people. [4, 5]

**1.3 TB control programme in Ethiopia**

In Ethiopia, about 60 - 80 % of health problems are because of communicable diseases and malnutrition. TB is among the leading causes of death and sickness in the country.[6] A well- organized TB control programme started in 1992 and the geographic coverage is 71%.[3]

Ethiopia ranks 7^th^ among high TB burden countries in the world. Ninety five percent of health institutions give DOTS service for population. Unfortunately, 40% of population do not have access to health service. The annual incidence and prevalence of all forms of TB is 356 and 533 per 10^5^ populations respectively. The case notification rate for all forms TB is 166 per 10^5^ populations. [4] The annual incidence of smear positive TB is 155 cases 10^5^ populations. The case notification of smear positive TB cases is 56 10^5^ populations. The case detection rate of all forms and smear positive cases is 47 % and 36 % respectively. The proportion of patients who are cured and completed treatment (treatment success rate) is about 76 %. [4]

**1.4. TB programme in southern region**

Southern Nations, Nationalities, and peoples Regional State (SNNPRS) is one of the Federal

States of Ethiopia. The region has a population of about 14 million. 93% of the population live in rural areas. The health service coverage and user rate is about 50 % and 32 %, respectively.

In 1995, DOTS started as a pilot project in three zones and four health institutions of the southern region of Ethiopia. Now, all hospitals and health centres provide DOTS to TB patients. As a result, number of TB cases has increased. However, the case detection rate was not in proportion to the programme coverage.[5] Low coverage of the health service, low use rate, and poverty compromised access to TB care. As a result, the case detection rate is below the global target; thus, we need to find better ways of addressing the TB problem.[5]

**2. Statement of the problem**

Ten years have passed since TB prevention and control programme was started in the southern region of Ethiopia. Experiences from the programme implementation for a decade showed the following findings. In the first five years, the programme was vertical and centralized with slow expansion. The case detection rate has increased compared with the existed TB programme coverage. However, many patients failed to adhere to treatment and follow up to give sputum specimen. The next five years there was better programme coverage and the cure and treatment completion rates has improved. However, the case detection remained low.

Currently the case detection rate of smear positive cases is only 39 %. The challenges were shortage of health workers, low health service coverage, low health service user rate, high disease burden, and socioeconomic barriers. Despite the challenges, TB prevention and control programme demands uninterrupted supplies, regular supervision, and strict adherence to DOTS. The low health service coverage compromises TB control. This gives opportunity for disease transmission and increases disease burden in the community. In addition, increasing the health service coverage and training health workers to fill the gap in short period seems a remote possibility. On the other side, improving health seeking behaviour and changing the low socio economic status would need longer period.

Therefore, TB control programme should find alternative ways that increase access to diagnostic and treatment service to TB patients. This will increase TB case detection and treatment. It may also decrease patient delays and lead to early initiation of treatment. This will decrease the risk of TB transmission in the community. This study aims at improving community based TB care in Ethiopia.

**3. Literature review**

Over the last two decades, because of the overlapping HIV AIDS pandemic, the number of active TB has increased in sub-Saharan Africa. This led to increased workload on the health services because of the dual epidemics. This needs major adjustment in TB control programme so patients could get better care. As a result, TB control programmes opted for different TB treatment supervision approaches. One of these was self-supervised therapy. In this alternative, patients receive drugs with out supervisory visit. Compared with facility-based TB care, self-supervised therapy improved treatment outcome. However, it needed intensive health education and regular patient supervision to increase adherence to treatment. In other studies, the treatment success rate was lower than institution based TB care. They self-supervised therapy in situations where direct supervision was not practical or was refused by the patients.[6]

Some studies adjusted the approach to improve patient adherence to treatment, namely modified DOTS. In this approach, they tried to reduce the number of TB patient visits to TB clinic. However, to compensate for reduced patient visits, health workers regularly visited patients and delivered health education to the patients. Sometimes, TB patients received incentives and enablers as meals and refunding transport costs so that patients will adhere to treatment. [7] This also leads to increased programme cost and workload on health workers. In addition, regular home visits by health workers encouraged TB patients’ adherence to treatment. This showed improved patient treatment success rate and decreased defaulter rates. However, maintaining regular health workers visits remained as a challenge. The study recommended motivation of health workers to attain better patient care. [8] However, this is difficult to achieve, as there is shortage of health workers. The problem is that without direct supervision of patients, the possibility of completion of treatment is unpredictable and low.

A consensus statement released by group of practitioners underlined that patient centred treatment strategy as a benchmark for TB control. The main reason is that it is more acceptable to the patient in his or her way of life than clinic or clinician centred treatment. In addition, it was more successful regardless of the country, community, or number of supervision carried out. Therefore, this needs organizing TB control programme in such a way that it will improve access, increase adherence to treatment and lessen socioeconomic burden. [7] And it should be within the existing community supported by a strong social and political network.[9]

Experience of community based TB care showed an improved TB treatment outcome in different settings. In Tanzania, community based TB treatment using guardian, as a treatment supervisor was as effective as facility-based TB treatment in urban settings. It showed that community based TB care is complementary to the conventional approach.[10] However, it was not possible to identify the margin between guardian and self-supervised treatment when it comes to direct treatment observation.

In Swaziland, they compared TB treatment supervision by CHWs and family members. They found that treatment supervision by family members was equally effective compared with supervision by CHWs. However, they recommended that selection of treatment supervisors should consider patient preference and access.[9, 11] As seen from above, the studies showed that community-based TB treatment is as effective as and could complement facility-based TB treatment. However, the variation of TB treatment supervisors from place to place remained a challenge to recommend the best supervisor under different settings.

On the other hand, improving TB case detection is one of the targets and main challenges of TB control. To solve this, investigators used different approaches to estimate the size of the disease in the community. TB prevalence surveys conducted used methods like home visits, small x-rays, and mobile diagnostic services. However, the number of TB cases detected was low in countries with low TB prevalence and the cost per case identified was too high to put into practice in poor countries.

WHO, as well as national TB control programme of Ethiopia, recommends ways to improve the case detection under the provision of self-reporting of patients to health facilities. These are public health education on early self- reporting to examination, training of health workers and making diagnostic facilities accessible to the patients.[3]

Improving the knowledge of general health workers will increase the index of suspicion of health workers to detect TB cases. In addition, it will improve the quality of information delivered to TB patients during treatment initiation and follow-up. In a study conducted in Tigray the relationship between the knowledge of CHWs and the health institutions mainly depends on the quality of care that the health institution is delivering. [12] This in turn might improve the health seeking behaviour of TB patients and hence case finding. Other investigators suggested symptom based TB screening in clinical and community setting when resources are limited [13-15]. Therefore, case detection requires simple and applicable community based TB case finding tools to detect cases as early as possible to deal with delayed presentation of patients to health institutions.

Late presentation of TB patients to medical care is still a major barrier to TB case finding. Study from southern Ethiopia showed the median delay in presentation to be about 4 months. Of these 75 % of patients had duration of illness more than two months.[16] In urban settings, the median patient delay was two months in addition to the health service delay of about one week. The delay is longer for patient living far away from health facilities and with low knowledge about TB. They recommended further decentralization of the service [17, 18]and introduction of feasible strategies that improve patient referral as early as possible. [19] Moreover, the association of poverty and stigma attached to TB played big role in increasing patient delay. So carrying out activities that focus on making TB care accessible to the poor and the rural community is important.[20]

Other reasons for delay are the inability of health workers to detect TB patients in the clinic. Therefore, understanding the main presenting symptoms of TB by health workers is important to increase TB case detection. Some studies identified the main symptoms described by TB patients. Specifically cough, night sweats, weight loss and tiredness are among the symptoms with high predictive value.[21, 22] A study conducted in Ethiopia described the main symptoms of TB patients and found that about 95 % had cough as a presenting symptom after two weeks.[23]

Health workers should have high index of suspicion of TB to increase case detection. This demands simple symptom score to improve diagnosis. This may reduce the duration of health service delay. This has been a practice for childhood TB case finding though it was not conclusive to date.[24] A study carried out in Ethiopia used TB symptoms and x-ray results to identify its diagnostic value. They found sensitivity and specificity of above 90%. However, about 40 % patients were below the cut-off point.[25] In addition, the cost and low access of radiological services in the country remains a practical problem to TB patients. Therefore, simple and applicable TB scoring system would play an important role in case finding by health and CHWs.

In addition, the nature of mycobacterium gives an opportunity to improve case finding. Mycobacterium TB remains viable for longer period when stored at a room temperature. A Study from Malawi showed that it could stay viable for about four weeks.[26] The fact that the bacilli could stay viable for longer time under room temperature in a humid set up could be an opportunity to TB control programme. Therefore, CHWs could collect and transport sputum specimen over short period for laboratory examination for TB bacilli.

Hence, the access to diagnostic health facilities might improve as well as the frequency of visits and cost incurred by the patients might decrease. One of the challenges to this approach is the non-adherence to the national diagnostic guidelines. [27] However, strengthening quality control system could minimize the problem. In addition, a study group in southern Ethiopia recommended regular on job training to laboratory technicians and regular supervision to improve the performance of laboratory technicians in peripheral diagnostic institutions. [28]

To be more pragmatic, implementation of TB control programme should consider the available inputs. In resource-constrained settings, programme managers opt for an intervention with low cost for improved programme indicators. Economic evaluations of TB control programme showed that treating TB is a cost-effective intervention. They further recommended additional effort to increase TB case finding and improve accessibility of service to the community in line with millennium development goals. [29]

Cost of community based TB care was determined in different countries. In Bangladesh, they compared the cost incurred by non-governmental organizations (NGOs) with that of the government for TB control programme. The NGOs used CHWs in TB case finding and patient follow-up. The cure rate was nearly equal in the two settings. However, the cost per TB patient cured by NGO was two thirds of government TB programme. The investigators recommended that collaboration with NGOs could improve TB care. However, the cost of sputum microscopy was higher than in the government programme.[30] They could also reduce the cost by strictly adhering to WHO recommendations for case finding.

In a study conducted in Uganda, they evaluated conventional hospital-based and community-based TB treatment for all TB patients. They admitted all smear positive TB patients in hospital for two months. Community based approach reduced the total cost per patient treated successfully by 43 % and increased success rate of smear positive TB patients by 19 %. The main drop in cost was because of the decreased duration of hospital admission from two months to nineteen days on average.[31] This mainly focused on hospital settings. Therefore, the cost of treating TB patients at health centre level needs further analysis.

An individual randomized controlled trial study conducted in Tanzania found that community-based TB treatment reduced cost per TB patient successfully treated by 35 % with similar treatment outcomes. The main cost drop was because of fewer visits to TB clinic. They recommended applicability of community based TB care in resource limited settings.[32] Before applying to a wider set-up, they should further evaluate the applicability at community level.

The World Bank reported that TB control programme as a cost-effective health intervention. [33] Different studies showed that community-based TB care is better than the existing conventional TB care. It also showed that it is less costly per patient treated and as effective or more compared to all alternative approaches [7, 34]. Therefore, this could be a better and feasible option in resource-constrained countries with high TB burden and overstretched health care.

In summary, community-based approach compliments the conventional facility based treatment. It minimizes cost per patients treated successfully and reduces the work burden on health workers. It is equally acceptable as health facility based treatment[35], improves patient adherence to treatment, and follow-up. Moreover, by incorporating simple of pulmonary TB, CHWs could assist in TB case finding and sputum collection and transport. Therefore, TB control programme should explore ways of improving community-based TB care in different settings.[36]

**4. Reason of the study**

Over the last two decades, the load of TB has increased in sub-Saharan Africa. The main reasons are human immunodeficiency virus infection and poverty. This led to an increased burden on the health services because of the associated morbidity and mortality. This led to health service exhaustion and forces patients to attend health institutions often.

The conventional passive case finding mostly serves those who have the access, knowledge, better health seeking behaviour and better socioeconomic status to go to health institutions. As TB patients are mostly from low socio-economic class, this will further lead to patient and health service delay and increase TB transmission in the community. In addition, getting access to health institutions for diagnosis and treatment is expensive and difficult for TB patients. Therefore, adherence and completion of full course anti-TB drugs remains a challenge to TB patients. This demands alternative approaches to improve access to diagnosis and treatment.

In Ethiopia, improving health service coverage and increasing the number of health workers to fill the gap seems a distant possibility. However, carrying out acceptable TB control in the community is important to complement the existing overstretched health service to improve health seeking behaviour and increase TB case finding. Therefore, using community-based approach to fill the gap in the TB control programme remains an unexplored alternative. So far, there has not been any study in southern Ethiopia to improve community based TB care. This study aims at improving community based TB care in southern Ethiopia.

**5**. **Goal of the study**

The goal of this research is to improve the TB control programme of Ethiopia

**5.1. General objective of the study**

To improve interventions to carry out community based TB control programme in southern Ethiopia.

**5.2. Specific Objectives:**

1. To estimate the case notification rate by involving CHWs in case finding,
2. To estimate the treatment outcome of TB patients supervised by CHWs

**6. Methods**

**6.1. Study area and population**

Southern region is the third large stand populous regions in Ethiopia. It is located in the southwest part of the country with a population of 14.08 million. It has thirteen zones and eight special districts. The health service coverage is 50 %. However, the user rate is only 32 %. TB diagnostic facilities are only located in health centres and less accessible to the patients.

Sidama zone is in southern region of Ethiopia. It has 10 districts and 2 city administrations. It is one of the most densely populated areas of the region with a population of about 2.8 million. It has one hospital, 20 health centres, 26 health stations, and 85 health posts. The health service coverage is 55%. In 1996, the DOTS started in Sidama. Currently all health facilities except all health posts deliver DOTS. The zone reports many TB cases. In addition, there are many CHWs in the zone. Some of these are community based reproductive health agents, traditional birth attendants, community health promoters, health extension workers and others. Practically so far, they have not participated in TB control programme.

**6.2.** **Study design**

This is a community-randomized trial where communities in the study area will be randomly allocated to intervention and control arms after obtaining consent from the community leaders.

**Part I Case finding**

Trained CHWs will be trained about how to organize sessions of community mobilization on monthly basis. They will teach about TB its prevention and control. CHWs from the intervention clusters will provide health education to the community and organize regular monthly sputum collection session at the health posts to suspects of pulmonary TB. They will use structured pretested questionnaire to identify suspects with symptoms suggestive of pulmonary TB (at least productive cough of two weeks or more) will give sputum specimen according to the recommendation of national TB guideline. The community health worker will keep the sputum specimen in a box prepared for this purpose and transport the sputum specimen, the same day, to diagnostic health institution for laboratory examination. The sputum specimens will be stained by a laboratory technician and will be examined by another technician (the technician will not know if the sputum was collected by CHWs or from the health institution) using by Ziehl-Neelsen technique on the same day. Then they will do direct microscopic examination and report the results. Patients diagnosed to have smear positive TB will start treatment under the direct supervision of CHWs. Patients in the non-intervention clusters will continue the conventional self-reporting and investigation in diagnostic health facilities. The study will follow the national TB programme guideline for patient diagnosis.

**Data collection techniques and tools**

As part of TB prevention and control strategy, CHWs will be trained how to identify TB suspects from the community. They will also receive practical training about using the diagnostic tool, sputum collection, and coding, labelling, and handling technique. TB programme will supply sputum cups and sputum collection boxes for CHWs. They will set up referral system among the health institutions, the laboratory technicians, and CHWs. In addition, will set dates for sputum transporting, smear result, treatment, and follow up of patients.

CHWs will organize sessions of community mobilization every month. All TB suspects who meet the criteria will give sputum specimen for laboratory examination. TB suspects will give sputum specimen as per the national guideline (spot-morning spot) CHWs will collect all sputum specimens, fill laboratory request formats, label sputum cups as per the national TB programme recommendation and keep it in a box prepared for sputum store in the community. CHWs will transport the sputum specimen and deliver it to the laboratory technicians in the diagnostic health institution.

The laboratory technicians will do three direct sputum microscopy examinations by using Ziehl-Neelsen technique on the same day when they received sputum specimen. Independent laboratory technician assigned (to receive sputum specimen and for staining) will stain the sputum specimen. Other laboratory technician with out knowing if it comes from the community or the health institution will examine the specimen. The results will be reported to the TB programme coordinator. The first technician who stained and blinded the specimen will also not know the result. Smear positive TB patients will start treatment in the community under the supervision of the community health worker as per the national TB programme recommendations.

**Part II Treatment outcome**

TB patients who are smear positive will be treated in the community under the supervision of CHWs as per the national guideline. CHWs will be oriented how to administer DOT by the health worker in the diagnostic institution. Follow up sputum examination will be conducted according to the national recommendation.

**Intervention sites**

The investigator, in collaboration with TB programme coordinators, will give training to health workers and CHWs about community based TB care and their role and responsibilities. The training will focus on TB case finding, case holding, drug management, recording and reporting as per the national guideline for community based TB prevention and control. The trainees will receive field guides to the trainees. Health workers will register TB patients on the unit TB registers provided by the national programme. CHWs will also register the patients on the daily treatment follow up format. Health workers will record TB case finding and treatment outcome of the intervention sites.

The regional TB control programme will supply all the needed drug and supplies to the respective districts and facilities via existing health care system. The scheme of supervision will have the following pattern: CHWs supervisors will supervise TB patients. Health workers will supervise the CHWs and the patients. Regular supervision as per the national recommendation will be conducted to the health institutions by the district programme coordinator. The investigator and coordinators at all levels - regional, zonal, and district will supervise health facilities, CHWs and the patients as the regional supervision scheme.

**Non-intervention sites**

TB patients in the non-intervention district will follow the existing facility based TB case finding and treatment. They will receive supplies and programme managers will carry out supervision according to the existing supervision system.

**Reporting**

District programme managers will report TB control activities according to the existing reporting system of the region as it carried out on quarterly bases. Reports include cases detected, treated, sputum follow up, drug, supervision laboratory supplies, and request for next quarter. In addition, they will submit research formats to the investigator. They will keep copies of their office.

**Data collection techniques and tools**

Health workers will register all the required information about TB patients as per the national recommendation and the study. At the end, health workers will hand over all records related to the study to the investigator. They will also report their activities to the health institutions, district, zone, and regional level. Data collectors will collect data from the reports and formats prepared for community-based TB care.

**Sample size calculation**

The sample size was calculated based on a difference in effect size of 30%, power of 80%, 95% significance level and coefficient of variation of 0·25. Based on the average annual smear positive TB case notification rate (CNR) of 64 per 10^5^ person years (unpublished review of three years of DOTS in the study area while the national CNR was 45 per 10^5^ person-years), we calculated the number of clusters required per group.

C = (Z1+Z2) ^2 * ((R1+R2)/Y + K^2(R1^2+R2^2))/ (R1-R2) ^2

C = number of cluster

n = the sample size from each group z_1_ =level of significance of 95 %( 1.96)

z_2_ =power of the study of 90 %( 1.28)

R_1_ = case notification rate in the intervention = 83.2 per 10^5^ population

R_2_ =case notification rate in the control = 64 per 10^5^ population

K= coefficient of variation = 0.25

Y = person year observation = 5000person-years

We calculated the number of clusters to be 24. Lastly based on the principle of allocating unequal number of cluster for randomization to increase power [37], we allocated 30 communities to the intervention and 21 communities to the control groups.

## 6.3. Permission to continue

The investigator will discuss with the national TB prevention and control programme about the procedure and all the supplies needed as drugs, reagents, sputum cups, and formats. In addition, the investigator will discuss with authorities and TB programme coordinators at all levels the regional health bureau, zonal, district, health institutions and the community leaders the aim and the implementation of the intervention study.

**6.4. Ethical clearance**

The investigator will get ethical clearance from Southern Nations, Nationalities, and Peoples Regional Health Bureau Ethical Review Committee. Suspects of plmonry TB that will be smear negative for acid fast bacilli will be encouraged to undergo medical examination for other lung disease. As per the national protocol smear negative suspects receive antibiotics. If suspects in the intervention sites deseve antibiotics will be encouraged to buy as per the decision of examining clinician. However, if they can not afford and produce poverty certificate the investigator will provide it.

**6.5. Data collection and handling**

In collaboration with programme coordinators, the investigator will train health workers and CHWs according to the training guideline. Except for those specifically prepared for the study, will use formats used by the national TB control programme formats. Training of data collectors will focus on community-based TB care activities, individual studies, data collection techniques, and administering a questionnaire. The training will have practical pretesting session. Data collectors will receive field guides and checklists prepared as a reference for the study period.

Health workers and CHWs will give questionnaires completed to the investigator or supervisors. The supervisors will check the completeness and accuracy of the questionnaires. The investigator will file and keep the filled questionnaires.

**6.6. Quality assurance**

CHWs will be trained about community based TB care, identifying TB suspects, sputum collection and handling, recording and reporting, selection of TB treatment supervisor, and drug and supply management. The data collectors will receive training materials, checklists, and field guides for field activities.

The research project and the national TB control programme will work together during the study period to help easy take over of the activity after the end of the study. Supervision schedule will be set in partnership with the follow-up plan of the health care system of the region except for specific supervision schedules for the intervention.

TB programme coordinators will supervise TB treatment supervisors during follow-up. The programme coordinator and the investigator will supervise the health institutions. In addition, the investigator, zonal and regional TB programme coordinators, will supervise data collectors and treatment supervisors.

Field supervisors will contact TB patients, CHWs, and health workers in the health institutions. They will crosscheck five per cent of the data in the community with TB unit register in health institutions and woreda TB registers. The programme coordinator alone, or with the principal investigator will supervise the activities and to provide supplies, do rapid assessment of the general activities, and give timely solution to the problems met. Centre for Health and Research laboratory will do the quality control for the slides examined in the diagnostic health institutions according to the national guideline and recommendation.

**6.7. Analysis plan**

The investigator will check the data, sort, and review it manually for errors and inconstancies. Then will use SPSS 13.1 for Windows for analysis as recommended for cluster level analysis.

# 7. Project management and work plan

The principal investigator, in teamwork with TB programme coordinators, local community leaders and CHWs, will oversee the TB control in the study areas. He will also take care of the administrative issues, overall help of the field activities and regular checking of the project progress. The principal investigator will keep proper records of the research and regularly communicate the progress and possible problems met with his supervisor.

**References**

1. Warring, F.C., Jr., *A brief history of tuberculosis.* Conn Med, 1981. **45**(3): p. 177-85.

2. M C Raviglione, A.P., *Evolution of WHO policies for tuberculosis control, 1948–2001.* THE LANCET, 2002. **359**: p. 775–80.

3. *Tuberculosis and Leprosy Prevention and Control Manual, Ministry of Health, Ethiopia.* 2002. **2nd Edition**.

4. *Global Tuberculosis Control Surveillance, Planning, Financing*. 2005, World Health Organization.

5. *Tuberculosis Leprosy and Blindness prevention and control program, Annual report*. 2004, Southern Nations, Nationalities and Peoples Regional Health Bureau, Awassa.

6. R Prasad, D.M.R., Surya Kant and A Jain, *A comparison of unsupervised treatment along with intensive health education and directly obsereved treatment in pulmonary tuberculosis.* Ind J Tub, 2001. **48**(21).

7. Chaulk, C.P. and V.A. Kazandjian, *Directly observed therapy for treatment completion of pulmonary tuberculosis: Consensus Statement of the Public Health Tuberculosis Guidelines Panel.* Jama, 1998. **279**(12): p. 943-8.

8. Jin, B.W., et al., *The impact of intensified supervisory activities on tuberculosis treatment.* Tuber Lung Dis, 1993. **74**(4): p. 267-72.

9. *WHO REPORT. Community TB care: Practice and Policy*. Vol. WHO/CDS/TB/2003.312. 2003.

10. Wandwalo, E., Kapalata, N., Egwaga, S., Morkve, O., *Effectiveness of community-based directly observed treatment for tuberculosis in an urban setting in Tanzania: a randomised controlled trial.* Int J Tuberc Lung Dis, 2004. **8**(10): p. 1248-54.

11. Wright, J., et al., *Direct observation of treatment for tuberculosis: a randomized controlled trial of community health workers versus family members.* Trop Med Int Health, 2004. **9**(5): p. 559-65.

12. Mengiste M Mesfin, T.W.T., Isreal G Tareke and Madeley RJ Richard., *Community health workers: their knowledge on pulmonary tuberculosis and willingness to be treatment supervisorsin Tigray, northern Ethiopia.* The Ethiopian Journal of Health Development, 2005. **Volume 19,**(Special issue, 2005,): p. 1-34.

13. Santha, T., et al., *Are community surveys to detect tuberculosis in high prevalence areas useful? Results of a comparative study from Tiruvallur District, South India.* Int J Tuberc Lung Dis, 2003. **7**(3): p. 258-65.

14. Golub, J.E., et al., *Active case finding of tuberculosis: historical perspective and future prospects.* Int J Tuberc Lung Dis, 2005. **9**(11): p. 1183-203.

15. A. K. Chakraborty, R.C., M.S. Krishina Murthy, A. N. Shashidhara, V. V. Krishina Murthy and K. Chaudhuri, *Prevalence of pulmonary tuberculosis in a peri-urban community of Bangalore under various methods of population screening.* Ind J Tub, 1994. **41**: p. 17.

16. Madebo, T. and B. Lindtjorn, *Delay in Treatment of Pulmonary Tuberculosis: An Analysis of Symptom Duration Among Ethiopian Patients.* MedGenMed, 1999: p. E6.

17. Demissie, M., B. Lindtjorn, and Y. Berhane, *Patient and health service delay in the diagnosis of pulmonary tuberculosis in Ethiopia.* BMC Public Health, 2002. **2**: p. 23.

18. Yimer, S., G. Bjune, and G. Alene, *Diagnostic and treatment delay among pulmonary tuberculosis patients in Ethiopia: a cross sectional study.* BMC Infect Dis, 2005. **5**(1): p. 112.

19. Mengiste M Mesfin, T.W.T., Isreal G Tareke, Yohannes T Kifle, Witten H Karen, and Madeley J Richard, *Delays and care seeking behavior among tuberculosis patients in Tigray of northern Ethiopia.* Ethiopian Journal of Health Development, 2005. **19**(special): p. 7 - 12.

20. Cambanis, A., et al., *Rural poverty and delayed presentation to tuberculosis services in Ethiopia.* Trop Med Int Health, 2005. **10**(4): p. 330-5.

21. G. Rathman, J.S., P. C. Hill, J. F. Murray, R. Adegbola, T. Corrah, C. Lienhardt, and K.P.W.J. McAdam, *Clinical and radiological presentation of 340 adults with smear-positive tuberculosis in The Gambia.* INT J TUBERC LUNG DIS, 2003. **7**(10): p. 942–947.

22. El-Sony, A.I., et al., *Symptoms in patients attending services for diagnosis of pulmonary tuberculosis in Sudan.* Int J Tuberc Lung Dis, 2003. **7**(6): p. 550-5.

23. Teklu, B., *Symptoms of pulmonary tuberculosis in consecutive smear-positive cases treated in Ethiopia.* Tuber Lung Dis, 1993. **74**(2): p. 126-8.

24. Mehnaz, A. and F. Arif, *Applicability of scoring chart in the early detection of tuberculosis in children.* J Coll Physicians Surg Pak, 2005. **15**(9): p. 543-6.

25. Tessema, T.A., et al., *An evaluation of the diagnostic value of clinical and radiological manifestations in patients attending the addis ababa tuberculosis centre.* Scand J Infect Dis, 2001. **33**(5): p. 355-61.

26. Banda, H.T., et al., *Viability of stored sputum specimens for smear microscopy and culture.* Int J Tuberc Lung Dis, 2000. **4**(3): p. 272-4.

27. Mengiste M Mesfin, T.W.T.a.M.J.R., *The quality of tuberculosis diagnosis in districts of Tigray region of northern Ethiopia.* The Ethiopian Journal of Health Development, 2005. **Volume 19**(Special issue, 2005,): p. 1-34.

28. Estifanos Biru Sharegie, M.A.Y.B.L., *Quality control of sputum microscopic examinations for acid fast bacilli in southern Ethiopia.* Ethiopian Journal of Health Development, 2005. **19**(2): p. 104 - 108.

29. Baltussen, R., K. Floyd, and C. Dye, *Cost effectiveness analysis of strategies for tuberculosis control in developing countries.* Bmj, 2005. **331**(7529): p. 1364.

30. Islam, M.A., et al., *Cost-effectiveness of community health workers in tuberculosis control in Bangladesh.* Bull World Health Organ, 2002. **80**(6): p. 445-50.

31. Okello, D., et al., *Cost and cost-effectiveness of community-based care for tuberculosis patients in rural Uganda.* Int J Tuberc Lung Dis, 2003. **7**(9 Suppl 1): p. S72-9.

32. Wandwalo, E., B. Robberstad, and O. Morkve, *Cost and cost-effectiveness of community based and health facility based directly observed treatment of tuberculosis in Dar es Salaam, Tanzania.* Cost Eff Resour Alloc, 2005. **3**: p. 6.

33. Musgrove, P., *Investing in health: the 1993 World Development Report of the World Bank.* Bull Pan Am Health Organ, 1993. **27**(3): p. 284-6.

34. Floyd, K., D. Wilkinson, and C. Gilks, *Comparison of cost effectiveness of directly observed treatment (DOT) and conventionally delivered treatment for tuberculosis: experience from rural South Africa.* Bmj, 1997. **315**(7120): p. 1407-11.

35. Wandwalo, E., et al., *Acceptability of community and health facility-based directly observed treatment of tuberculosis in Tanzanian urban setting.* Health Policy, 2005.

36. Sinanovic, E., et al., *Cost and cost-effectiveness of community-based care for tuberculosis in Cape Town, South Africa.* Int J Tuberc Lung Dis, 2003. **7**(9 Suppl 1): p. S56-62.

37. Donner A, Klar N (2000) Design and analysis of cluster randomization trials

in health research. London: Arnold.

.

**Community consent form Improving community based TB care in southern Ethiopia**

Request of participation

Ethiopia is among high TB burden countries in the world. As a result, our region shares similar problem. The conventional method of treating TB mainly focuses on health institution based directly observed treatment. TB patients travel to health facilities on daily bases to get the treatment. This costs them extra time and money they expend for diagnosis and treatment.

Our study aims at improving community-based TB care by making the service available in the community whereby CHWs treat the patients. The advantage of the intervention is that patients will not be visiting health facilities daily. This will in turn decrease the related expenses and improve adherence.

TB patients in the community will have equal access to the diagnostic and treatment facilities as other patients in the region. There is no obligation and related punishment in case you do not like to take part in the study. Your participation in the study is fully based on voluntary decision. You have the right to participate and withdraw from the study. Every patient in the community will get the detail information about the intervention and the decision will be left for them as to participate or not in the study.

In case of inconveniences or for more information, Dr. Daniel Gemechu will be available during supervision and you could contact him through health workers in the community and use the following address.

Box 303 Awassa,

Tele. 00 251 46 2202847

In addition, you have the right to ask CHWs supporters, health professionals in the

health institutions and district programme managers.

**TUBERCULOSIS SYMPTOM BASED SCREENING TOOL**

**Questionnaire no.________ Name of interviewer ______________________Date__________**

**1. Socio-demographic variables**

1.1 Name of suspect _______________________________ 1.2. Age_____1.3. Sex ___________

1.4. Cluster __________ Kebele ______________

1.5. Marital status Single___ Married___ Divorced____Widowed___ other (specify) __________

1.6. Educational status No schooling ______ Grade ______ other (specify) ________________

1.7. Occupation of suspect farmer _ student _ merchant ___ housewife __gov employee___ others (specify) __

**2. Tuberculosis symptoms and history**

|  | Tuberculosis symptoms and history | No | Yes | Duration in weeks |
| --- | --- | --- | --- | --- |
| 2.1. | Did you experience cough for two or more weeks? |  |  |  |
| 2.2 | Is the cough productive of sputum? |  |  |  |
| 2.3. | Does sputum contain blood? |  |  |  |
| 2.4. | Did you have fever? |  |  |  |
| 2.5. | Did you have loss of appetite? |  |  |  |
| 2.6. | Did you loss weight? |  |  |  |
| 2.7. | Did you have chest pain? |  |  |  |
| 2.8. | Did you have history of tuberculosis treatment? |  |  |  |
| 2.9. | Did you have closer contact with known tuberculosis patient? |  |  |  |

**CURRICULUM VITAE**

**1. Personal information**

Full Name: Daniel Gemechu Datiko Date of Birth: February 20/1973

Sex: Male Place of Birth: Gidole, Ethiopia

Marital Status: Married Nationality: Ethiopian

Religion: Protestant

**2. Educational Background**

- Doctor of Medicine (MD) from Addis Ababa University (1993 - 1999)

**3. Work Experience**

- July 2003 - July 2005: Regional TB Leprosy and Blindness programme coordinator
- January - July 2003 : Yirgalem Hospital Sidama Zone, Assistant Medical Director
- April 2001 - July 2003 : Yirgalem Hospital Sidama Zone, General medical Practitioner
- October 1999 - April 2001 : Darara Health Centre, General medical Practitioner

**5. Language ability**:

1. Amharic - Reading , Writing and Speaking
2. English - Reading , Writing and Speaking

**6. Professional Membership**

- Member of Ethiopia Public Health Association (EPHA)

**7. Professional Interest**

- Tuberculosis prevention and control
- Community based interventions

**8. References**

- Dr Shiferaw Tekle MariamHabtamu, Head of regional Health Bureau, Awassa

[shiferaw_t@hotmail.com](../shiferaw_t@hotmail.com), [t_kmariam@yahoo.com](../t_kmariam@yahoo.com), Tele: +251 46 2203263

**9. Address**

University of Bergen, Centre for International Health, Armeur Hanson Building,

Box 5023, Bergen, Norway

E-mail: [Gemechu.Daniel@student.uib.no](../Gemechu.Daniel@student.uib.no%20)

Po Box: 303

Awassa, Ethiopia

Tele: Res: +251 46 2202847

E-mail: [danieljohn42@yahoo.com](../danieljohn42@yahoo.com%20)

I hereby notify that, to the best of my knowledge and ability the information given above is genuine and true.

Daniel Gemechu, 2006
